# Essential Oils in Respiratory Mycosis: A Review

**DOI:** 10.3390/molecules27134140

**Published:** 2022-06-28

**Authors:** Mónica Zuzarte, Lígia Salgueiro

**Affiliations:** 1 Faculty of Medicine, Coimbra Institute for Clinical and Biomedical Research (iCBR), University of Coimbra, 3000-548 Coimbra, Portugal; 2 Center for Innovative Biomedicine and Biotechnology (CIBB), University of Coimbra, 3000-548 Coimbra, Portugal; 3 Clinical Academic Centre of Coimbra (CACC), 3000-548 Coimbra, Portugal; 4 Faculty of Pharmacy, University of Coimbra, 3000-548 Coimbra, Portugal; ligia@ff.uc.pt; 5 Faculty of Sciences and Technology, Department of Chemical Engineering, Chemical Process Engineering and Forest Products Research Centre (CIEPQPF), University of Coimbra, 3030-790 Coimbra, Portugal

**Keywords:** aspergillosis, candidiasis, cryptococcosis, endemic infections, opportunistic infections, plant volatiles

## Abstract

Respiratory mycosis is a major health concern, due to the expanding population of immunosuppressed and immunocompromised patients and the increasing resistance to conventional antifungals and their undesired side-effects, thus justifying the development of new therapeutic strategies. Plant metabolites, namely essential oils, represent promising preventive/therapeutic strategies due to their widely reported antifungal potential. However, regarding fungal infections of the respiratory tract, information is disperse and no updated compilation on current knowledge is available. Therefore, the present review aims to gather and systematize relevant information on the antifungal effects of several essential oils and volatile compounds against the main type of respiratory mycosis that impact health care systems. Particular attention is paid to *Aspergillus fumigatus*, the main pathogen involved in aspergillosis, *Candida auris*, currently emerging as a major pathogen in certain parts of the world, and *Cryptococcus neoformans*, one of the main pathogens involved in pulmonary cryptococcosis. Furthermore, the main mechanisms of action underlying essential oils’ antifungal effects and current limitations in clinical translation are presented. Overall, essential oils rich in phenolic compounds seem to be very effective but clinical translation requires more comprehensive in vivo studies and human trials to assess the efficacy and tolerability of these compounds in respiratory mycosis.

## 1. Introduction

Respiratory infections have increased over the past decades, becoming important causes of morbidity among immunocompromised and immunosuppressed patients and accounting for 4.3 million annual deaths worldwide [1]. Among these, fungal infections are underestimated, despite being responsible for mortality rates above 50% [2]. Although exposure to these respiratory pathogens occurs regularly during a lifetime, healthy individuals rarely develop symptomatic infections. However, in individuals with impaired defenses—for example, due to the use of chemotherapeutic and immunosuppressive agents, and antibiotics, or having prosthetic devices, grafts, burns, neutropenia or HIV—systemic life-threatening fungal infections may occur [3].

Overall, two main types of infections can occur: endemic (primary) or opportunistic. The first occur primarily in patients living or traveling in developing countries and, despite being mostly asymptomatic or causing mild infections, they account for huge health-care costs in these regions. Importantly, an exposure to high concentrations of inoculum or in patients with compromised immune defenses, life-threatening infections or the reactivation of latent foci may occur. The most common fungal infections include blastomycosis, coccidioidomycosis, histoplasmosis, paracoccidioidomycosis and sporotrichosis [4]. On the other hand, opportunistic infections generally occur in debilitated patients with impaired defense mechanisms and have a global distribution. These include aspergillosis, candidiasis, cryptococcosis, hyalohyphomycosis, mucormycosis, phaeohyphomycosis, and *Pneumocystis* pneumonia, with *Aspergillus*, *Cryptococcus* and *Pneumocystis* being the major pulmonary fungal pathogens capable of causing life-threatening invasive diseases [5]. In Section 2, a detailed characterization of these infections is presented with information regarding diagnosis, pathological symptoms and treatment.

The diagnosis of respiratory mycosis is quite difficult as it may present nonspecific symptoms and noninvasive diagnostic tests are poorly sensitive. Therefore, a combination of factors is frequently considered for an accurate diagnosis, namely clinical setting, chest imaging, and negative bacterial or viral studies [6]. To treat respiratory mycosis, several antifungals are available and include triazoles, echinocandins and formulations with amphotericin B. The first interfere with cytochrome P450 (CYP450) and lead to the inhibition of lanosterol, thus decreasing ergosterol synthesis and inhibiting cell-membrane development [7]. The most common are fluconazole and itraconazole, which are effective against *Candida* (except *C. glabrata* and *C. krusei*), *Cryptococcus*, *Blastomyces*, *Histoplasma*, and *Coccidioides*. The second-generation triazoles, including voriconazol and posaconazole, are also active against *Aspergillus* and *Mucor*. As these drugs undergo hepatic metabolism, drug–drug interactions may occur through the CYP450 system and side effects such as gastrointestinal disturbances, hepatotoxicity, headache, and rash have been reported [8]. Echinocandins induce osmotic instability and cell death in fungi, by compromising the synthesis of beta-(1,3) glucan synthase, an important component of their cell walls [7]. Examples of these antifungals are caspofungin, micafungin and anidulafungin, primarily used against *Candida*, *Aspergillus* and *Pneumocystis*. Treatment with echinocandins can cause rash, headache, fever, and chills [9]. Amphotericin B (AmB) also causes fungal death by creating channels or pores on the fungal cell membrane that leak cellular components [10]. Side-effects include weight loss, headache, fatigue, and phlebitis and most importantly nephrotoxicity that can be mitigated by using amphotericin B (AmB) formulations such as AmB colloidal dispersion and lipid formulations. Furthermore, combination therapy has also been considered as it tends to increase treatment efficacy, especially for drug-resistant fungal isolates [11]. Indeed the emergence of resistance is another challenging situation that has increased over the decades due to the use of azole fungicides for agricultural purposes [12]. Therefore, natural antifungal alternatives have emerged as promising agents to overcome resistance concerns and unwanted side-effects. The present review provides an updated compilation of the studies performed in the last 20 years based on a bibliographic search conducted using PubMed, Scopus and Google Scholar databases.

## 2. Types of Fungal Infections of the Respiratory Tract

Respiratory infections can be caused by the inhalation of spores from fungi that inhabit the environment. Although most people have been exposed to these pathogens, symptoms are rare in healthy individuals and infections are generally not transmitted between humans. However, in immunocompromised individuals these infections are of major concern being *Aspergillus*, *Cryptococcus*, *Pneumocystis*, and endemic fungi the major pulmonary fungal pathogens able to cause life-threatening invasive diseases [5]. Moreover, several risk factors, such as prolonged antibiotic use, hematologic malignancies and other immunocompromised states, worsen infection outcomes. Although the number of known fungi is relatively high—around 70,000—only 100 have been detected in respiratory infections with only a few being consistently considered pathogenic. Several of these fungi present dimorphism, with primary pathogens and *Sporothrix schenckii*, showing a morphological transformation in host tissues from a hyphal form to a yeast-like form (or spherule in the case of *Coccidioides immitis*). Contrarily, *Candida albicans* alters from blastoconidia to germ tubes that further develop into hyphae and *Penicillium marneffei* converts to transversely dividing sausage-shaped cells [13]. Moreover, many of these pathogens are capable of biofilm growth, forming highly organized communities that are resistant to antifungals and are responsible for recurrent infections [14]. Table 1 summarizes the main fungi responsible for relevant respiratory mycoses and points out for each pathogen (divided into endemic or opportunistic) the main source of infection, relevant pathological manifestations, diagnostic methods, and current therapy. The majority of information on some of the pathogens included in the table was based on the online textbook *Microbiology*, an OpenStax resource [15], the information on the remaining fungi being completed with other bibliographic sources such as clinical guidelines [16], centers for disease and health control [17] and professional manuals [18] available online.

## 3. In Vitro and In Vivo Models to Assess Antifungal Properties

The search for new antifungal agents involves in vitro susceptibility tests. Reference methods, namely the Clinical and Laboratory Standards Institute (CLSI) and the European Committee on Antimicrobial Susceptibility Testing (EUCAST) standard methods, are available enabling more practical, reliable, and reproducible protocols for antifungal susceptibility testing. Both resort to the broth microdilution method and assess the minimal inhibitory concentration (MIC) of an antifungal drug, which indicates the minimal drug concentration that inhibits fungal growth. Although these tests present some slight methodological differences, MICs are comparable [19]. Commercial antifungal susceptibility tests are also available. Examples include broth microdilution methods such as the Sensititre^TM^ or YeastOne^TM^ that use color endpoints based on metabolic dyes such as AlamarBlue, incorporated into the growth media or agar-based methods, such as the Etest^®^ and the automated system VITEK^®^ 2 [20].

In addition to in vitro testing, pre-clinical animal models have enabled us to increase the knowledge on fungal infections and putative therapeutic strategies. In these assays it is important to consider the model species and its immune status, the route of infection, and the fungal strain used, as these aspects can impact experimental outcomes. Overall, inbred strains of laboratory mice are the most common models mainly due to their relatively low cost and the wide availability of immunological reagents. However, other vertebrates such as rats, guinea pigs, rabbits, and zebrafish have gained popularity [21]. Recently, the *Galleria mellonella* model has been one of the most used as it is inexpensive, easy to use, and does not require a dedicated infrastructure, the antifungal efficacy of the drug being estimated by fungal burden or mortality rate in infected and treated larvae [22]. To mimic respiratory mycosis, an intranasal or intratracheal injection of a liquid fungal suspension or the inhalation of dry fungal cells can be used. A catheter can be inserted beyond the vocal cords to facilitate intratracheal delivery of fungal cells and their dispersion into the pulmonary parenchyma may be enhanced by mechanical ventilation [23] or by using a microsprayer attached to the syringe tip [24]. Classical readouts include organ fungal burden and histopathology.

## 4. The Relevance of Essential Oils

Essential oils are mixtures of volatile compounds present in various organs of aromatic plants and obtained from the plant by hydrodistillation, steam distillation or dry distillation or, in the case of *Citrus* fruits, by a suitable mechanical process [25,26]. Essential oils generally present a strong odor and high lipophilicity, being primarily composed of terpenic compounds, such as monoterpenes and sesquiterpenes. In some cases, phenylpropanoids may also occur in high amounts and more rarely nitrogen and sulphur derivatives can be found. An important aspect to consider is their high chemical variability mainly due to environmental factors or genetic variations [27]. The latter may result in the expression of different metabolic pathways with significant variations in essential oils’ composition that justify the identification of chemotypes. To assure high quality of the commercialized essential oils, analytical guidelines published by several institutions, such as the European Pharmacopoeia, the International Standard Organization (ISO), and the World Health Organization (WHO), should be followed.

Essential oils play relevant natural roles primarily in plant defense and signaling processes and have been explored by several industries due to particular features such as aroma, taste and bioactive potential. Indeed, they are valuable raw materials in the pharmaceutical, agronomic, food, sanitary, cosmetic, and perfume industries [28]. In what concerns bioactive potential, several studies have highlighted the antifungal and anti-inflammatory potential of these compounds as reviewed elsewhere [29,30]. These properties are quite relevant in the context of respiratory mycosis as fungal infections are associated with acute inflammation, which exacerbates the infection and delays its eradication. Therefore, extracts or compounds that combine both antifungal and anti-inflammatory activities, at concentrations devoid of toxicity, emerge as suitable preventive/therapeutic agents.

### 4.1. Antifungal Effect of Essential Oils on Respiratory Mycosis

Essential oils and their volatile compounds have shown promising effects against fungi involved in infections of the respiratory tract. Overall, *Aspergillus* spp., *Candida albicans*, and *Cryptococcus neoformans* are by far the most assessed strains, with only a few studies being carried out on other relevant pathogens referred in Table 1. Next, a compilation of selected studies is presented, the assortment criteria being defined in each section.

#### 4.1.1. Essential Oils in Aspergillosis

Regarding *Aspergillus* pathogens, *A. fumigatus* is the most ubiquitous strain and the major causal agent of aspergillosis, followed by *A. flavus*, *A. niger*, *A. terreus*, and *A. nidulans* [31]. Besides species diversity, several types of aspergillosis are known, namely pulmonary aspergillosis that generally develops in patients with underlying lung pathology; allergic bronchopulmonary aspergillosis, an allergic reaction that results from hypersensitivity to *Aspergillus* colonization and is generally exclusive to asthma and cystic fibrosis patients and invasive aspergillosis, the most severe type that occurs when the infection travels from the lungs into the bloodstream [32]. As *A. fumigatus* is the main pathogen involved in aspergillosis, a compilation of the studies showing minimal inhibitory (MIC) or minimal fungicidal/lethal concentrations (MFC/MLC) is presented in Table 2. Only essential oils presenting MICs lower than 10 mg/mL were considered, being the plant name, part of the plant used to obtain the essential oils and antifungal effect highlighted, whenever this information was available in the original study. Broader studies, gathering more than three species, are not included in the table, and are discussed next. Furthermore, since only a few studies assessed a possible mechanism of action underlying these effects, this topic is discussed in Section 4.2.

As previously mentioned, some studies have included several essential oils. For example, in a study involving fifteen species from the Asteraceae family, only *Achyrocline alata* and *Baccharis latifolia* essential oils were effective against *A. fumigatus* (geometric mean MIC value of 78.7 and 157.4 µg/mL, respectively) [62]. Moreover, in some cases both essential oil direct contact or vapor phase are assessed, thus foreseeing different modes of administration and distinct applications. For example, resorting to these two strategies, the essential of *Eugenia caryophyllata*, *Lavandula angustifolia*, *Origanum vulgare*, *Salvia sclarea*, *Thuja plicata* and *Thymus vulgaris* were assessed, being *Origanum vulgare* essential oil the most effective, through direct contact, with a MIC value of 0.025%. However, the volatile vapor of 0.075% of the majority of the oils was also able to exert a fungicidal effect on *A. fumigatus*, proving that these oils can be used, for example, for environmental disinfection [63].

Synergistic studies between essential oils and isolated compounds or essential oils with conventional antifungals are also relevant, thus reducing the effective dose needed and improving therapeutic outcomes [64]. Indeed, a chessboard assay using twenty-five essential oils showed that a combination of *Cymbopogon citratus* and *Thymus serpyllum* oils was the most potent against *A. fumigatus* (fractional inhibitory concentration index -FICI- of 0.1875; with a total synergistic effect considered for values ≤ 0.5) [65]. Additionally, *Thymus vulgaris* essential oils and thymol showed significant levels of synergistic interaction with fluconazole presenting an FICI value of 0.187 [40].

Moreover, mixed microbial infections are known to cause significant health care burdens and are commonly found in patients with chronic infections. Therefore, some studies have also considered this reality by assessing the polymicrobial antibiofilm effect of essential oils. A study carried out by Pekmezovic and colleagues addressed this ability using selected *Citrus* essential oils and showed that 10 mg/L of the oils were able to both inhibit a mixed biofilm formation composed of *Pseudomonas aeruginosa* and *Aspergillus fumigatus* and affect quorum sensing [66].

Finally, in vivo validations are of utmost importance but are quite sparse regarding respiratory mycosis. A pre-clinical study confirmed the potential of *Leptospermum petersonii* essential oil by showing a significant reduction of fungal infection in the lungs of animals that completed the treatment regimen and the effect was strikingly superior to that reported for conventional antifungal drugs [48].

Regarding the potential of isolated compounds, very few studies have been performed. Once again phenolic compounds stand out as very effective, namely eugenol—MIC = 250 ug/mL [49], thymol—MIC = 192 μg/mL [40] and carvacrol—MIC = 0.16 µL/mL [51]. Indeed, this could explain the excellent activity of some of the essential oils pointed out above such as *Thymus vulgaris* (rich in thymol) and *Lavandula multifida* (with high amounts of carvacrol). Besides phenolic compounds, other essential oils constituents such as safrole have shown very effective antifungal abilities against *A. fumigatus*, with a MIC of 39.1 µg/mL [42]. Interestingly, although the essential oil obtained from the roots of *Cinnamomum camphora* had a high concentration of this compound (57.6%), the antifungal potential of the whole extract was not so prominent (MIC = 312.5 µg/mL), thus showing that, in some cases, antagonistic effects may also take place between different compounds present in the mixture. Additionally, 1,8-cineole, a compound widely found in many essential oils, showed promising effects with a MIC value of 156.3 µg/mL [42].

#### 4.1.2. Essential Oils in Respiratory Candidiasis

*Candida* infections are quite difficult to detect as *Candida* spp. inhabit the normal microflora of the skin, oral cavity, gastrointestinal mucosa, respiratory tract, and genitourinary tract [67]. Moreover, despite covering more than two-thirds of fungal infections, invasive candidiasis (defined as the presence of *Candida* in the blood—candidemia—or firmly established *Candida* infections) rarely manifest as *Candida* pneumonia and antifungal treatments in these cases is a matter of ongoing debate [68]. Nevertheless, *Candida albicans*, *C. glabrata*, *C. tropicalis*, *C. parapsilosis* and *C. krusei* have been pointed out as the most prevalent strains in invasive candidiasis. Since several reviews have largely covered the antifungal potential of essential oils on these pathogens [69,70,71,72,73], we now focus on a once considered rare species, *Candida auris*, that is currently emerging as a major pathogen in certain parts of the world. *C. auris* infections are difficult to treat as this pathogen does not respond to conventional antifungal drugs. This fungus also presents a high risk of transmission as it persists on several equipment and medical devices [74]. Importantly, some essential oils have shown promising antifungal potential against this species. Table 3 compiles this information by pointing out the plant species, part of the plant used to obtain the essential oil and its main compounds, whenever referred to in the original study. The antifungal potential of the essential oil is also highlighted, as well as the mechanism of action underlying the observed effect, if assessed.

Although *C. auris* is an emergent species, several studies have already shown the potential of essential oils as effective antifungal agents, as highlighted in Table 3. Nevertheless, as different susceptibility tests are used and units lack uniformity among studies, it is quite difficult to perform comparisons between studies and understand which essential oil is the most effective. Even so, it seems that essential oils rich in phenolic compounds exert a more potent inhibitory effect. Indeed, the phenolic compound carvacrol assessed alone has already shown promising effects. A median MIC of 125 µg/mL was reported as well as synergistic and additive effects in combination with the conventional antifungals fluconazole, amphotericin B, nystatin and caspofungin [82].

#### 4.1.3. Essential Oils in Cryptococcosis

Pulmonary cryptococcosis, contrarily to cryptococcal meningitis, remains underdiagnosed mainly due to limitations in diagnostic tools. Indeed, the infection presents similar clinical and radiological features to lung cancer, pulmonary tuberculosis, bacterial pneumonia, and other pulmonary mycoses. The genus *Cryptococcus* consists of more than 70 species, the two main human pathogens being *C. neoformans* and *C. gattii* [83]. Several studies have also pointed out the antifungal potential of essential oils, mainly for *C. neoformans*. Table 4 compiles these studies referring to the plant name, part of the plant used to obtain the essential oils, and antifungal effect. Since the mechanisms of action underlying these effects are poorly explored, this topic will be discussed collectively in Section 4.2.

Besides the studies referred to in Table 4, others were performed gathering a higher number of species, with bigger data approaches enabling more extensive analysis. For example, using this approach, eighty-two essential oils were analyzed, with fifteen being highlighted as very potent (MIC ≤ 100 μg/mL), and from these *Cedrus atlantica* standing out as the most effective, with a MIC value of 20 μg/mL [116]. In another extensive study, the antifungal potential of sixty commercially-available essential oils was also assessed, with *Cinnamomum cassia*, *C. zeylanicum*, *Coriandrum sativum*, *Pogostemon cablin*, *Santalum album*, *S. austrocaledonicum*, *S. paniculatum* and *Vetiveria zizanoides* being very effective (MIC = 20 µg/mL) [117].

Some studies also resort to ethnopharmacological evidence and tend to assess the potential of essential oils based on their traditional uses, thus recovering relevant knowledge that tends to be lost over time. For example, the study carried out by Lawson and colleagues considered a specific group of plants from the Asteraceae family used in Cherokee and other Native American traditional medicines, and showed a very potent effect of *Eupatorium serotinum* essential oil against *Cryptococcus neoformans* with a MIC value of 78 μg/mL [118].

Regarding synergistic studies between essential oils and conventional drugs, interesting results against *Cryptococcus neoformans* have also been reported. For example, Scalas and colleagues, through checkerboard testing and isobolographic analysis, showed synergistic effects (FICI ≤ 0.5) between itraconazole and *Origanum vulgare*, *Pinus sylvestris* or *Thymus vulgaris* essential oils. Importantly, a synergistic effect was also observed with itraconazole and *Thymus vulgaris* essential oil (chemotype: thymol 26.52%; carvacrol 7.85%) on an azole not susceptible strain of *Cryptococcus neoformans*, thus confirming the potential of essential oils as cost-effective adjuvants in antifungal therapy [119]. Using similar methodology, the combination of *Mentha* x *piperita* essential oil with itraconazole also exerted a synergistic effect (FICI ≤ 0.5), with a decrease in the MIC. Nevertheless, on the azole-resistant strain, the binary combination of itraconazole and the oil yielded additive effects [97]. Furthermore, the combination of *Ocimum basilicum* var. Maria Bonita (a genetically improved cultivar) essential oil with fluconazole enhanced the antifungal activity, especially against the resistant strain of *Cryptococcus neoformans*, with MIC being reduced from 1250 μg/mL to 625 μg/mL [120].

Combinations between essential oils have also shown positive effects on *Cryptococcus neoformans*. For example, combining the essential oils of *Boswellia rivae*, *B. neglecta* and *B. papyrifera* with *Commiphora guidotti* or *C. myrrha* oils displayed synergistic, additive and noninteractive properties with MICs ranging from 0.5–5.3 mg/mL [121].

Regarding isolated compounds, several studies have been performed, showing that the most potent compounds against *Cryptococcus neoformans* were (E)-Dec-2-enal with a MIC of 25.65 ug/mL [85], α-pinene with a MIC value of 0.07 mg/mL [113], and (R)-(+)-limonene with a fungicidal effect at 0.08µL/mL [112]. These effects may explain the potential of *Aristolochia delavayi* essential oil, with high amounts of (E)-dec-2-enal [85], and *Bupleurum rigidum* subsp. *paniculatum* and *Thapsia villosa* rich in α-pinene [88,112]. Geraniol also showed promising effects with a study pointing out a MIC of 76 µg/mL [120]; nevertheless in other studies, the MICs were quite distinct (MIC_80_ = 128 µg/mL [122] and MIC = 0.32 μL/mL [61]). Variability between MIC values can occur mainly due to variations between laboratories and strains [123]. Other compounds showed some antifungal potential but to a less extent, namely linalool (MIC = 5 μL/mL) and terpinen-4-ol (MIC = 1.25 μL/mL) [61], sabinene (MIC = 0.32μL/mL) and *cis*-*β*-ocimene (MIC = 0.16–0.32μL/mL) [102], methyleugenol (MIC= 0.32 μL/mL) [112], and carvacrol (MIC= 0.16 μL/mL) [51].

#### 4.1.4. Essential Oils on Other Respiratory Infections

Concerning other fungal strains involved in respiratory mycosis, namely those referred to in Table 1, in vitro studies have been performed, although to a much lesser extent. Table 5 compiles these studies and points out their main findings.

Regarding isolated compounds, several studies have been performed. Overall, farnesol, a sesquiterpenic compound, and terpinene-4-ol, a terpene alcohol, were the most assessed and showed very promising effects. For example, farnesol was tested against *Coccidioides posadasii*—MIC = 0.002–0.01 mg/L [127], *Histoplasma capsulatum* var. *capsulatum*—MIC = 0.008–0.003 µM [128] and *Sporothrix schenckii*—MIC = 0.003 to 0.222 μg/mL [129]. Importantly, in the first two pathogens, synergistic effects with amphotericin B, itraconazole, voriconazole and caspofungin or itraconazole, respectively were observed [127,128], thus confirming its potential as an adjuvant in fungal infections. On the other hand, terpinene-4-ol was tested against *Sporothrix schenckii* (MIC = 4–32 mg/L) and was able to decrease its cellular ergosterol content. In combination with itraconazole or terbinafine, it also exerted a synergistic effect [130]. Furthermore, this compound was also effective against *Coccidioides posadasii* (MIC = 350–1420 μg/mL) and *Histoplasma capsulatum* [MIC = 20–1420 μg/mL (filamentous phase) and 40–350 μg/mL (yeast phase)], although to a lesser extent [131].

Other compounds have also been tested, such as the monoterpene p-cymene (MIC = 1024 μg/mL) and the phenolic compound thymol (MIC = 128–256 μg/mL), against *Rhizopus oryzae* [124]. Furthermore, the sesquiterpene dialdehyde polygodial was also very effective against *Penicillium marneffei* with a MIC value of 3.3 μg/mL [132]. These studies, although still limited in number, show that other fungal pathogens are being considered for antifungal susceptibility testing, besides the gold standard strains, thus opening new avenues for the development of new preventive/therapeutic strategies.

### 4.2. Mechanism of Action Underlying Essential Oils Antifungal Effects

The majority of the in vitro studies performed tend to identify the MIC and MLC/MFC of the volatile extract/compound and in some cases, the putative mechanism of action underlying the antifungal effect. Indeed, several methods have been proposed to elucidate the target site or the mechanism of action of the essential oils, in fungal cells [133]. Generally, mechanistic studies are performed on *Candida albicans* and the main effects considered are biofilm disruption, cell morphology and plasma membrane integrity. In less extent mitochondrial enzymes, reactive oxygen species (ROS) and gene expression are also assessed. Next, studies performed on other pathogens rather than *Candida albicans* are highlighted, namely for biofilm and cell wall/membrane integrity.

Biofilms are important virulence factors for pathogenic fungi naturally formed when fungi change from a planktonic to a sessile state and attach to surfaces and to each other, being involved and protected by a polymeric extracellular matrix. The fungi also secrete quorum-sensing molecules that play a relevant role in fungal resistance and pathogenicity [134] and, therefore, constitute a very promising therapeutic target.

Kumari and colleagues carried out a study using several compounds present in essential oils and confirmed their anti-biofilm effect on *Cryptococcus laurentii* and *Cryptococcus neoformans* in the following order: thymol > carvacrol > citral > eugenol = cinnamaldehyde > menthol. Indeed, for the most effective compounds, a potent effect on biofilm morphology was confirmed by scanning electron microscopy and confocal laser scanning microscopy that showed the absence of extracellular polymeric matrix, reduction in cellular density and alteration in the surface morphology of biofilm cells [122].

Other relevant therapeutic targets that are highly assessed are the structural elements of fungi cell walls and membranes, as their inhibition can affect cell wall maturation, septum formation, and bud ring formation, by damaging cell division and cell growth. Essential oils are able to disrupt the cell wall, leading to cytoplasm leakage and compromise fungi membrane permeability and fluidity by altering its properties and compromising membrane-associated functions. Indeed, morphological and ultrastructural alterations were observed in *Aspergillus fumigatus* exposed to *Cuminum cyminum*, *Nigella sativa* and *Ziziphora clinopodioides* essential oil that interfered with the enzymes involved in cell wall synthesis, caused high vacuolation of the cytoplasm, detachment of the fibrillar layer of cell wall, and plasma membrane disruption. Additionally, disorganization of nuclear and mitochondrial structures was observed [44]. Similarly, *Leptospermum petersonii* essential oil disturbed *Aspergillus fumigatus* cell membrane with alterations observed in hyphal morphology, susceptibility of spheroplasts and uptake of propidium iodide following exposure to the oil [48]. Regarding isolated compounds, eugenol reduced the cell diameter and capsule size of *Cryptococcus gattii* and *C. neoformans*. The compound was also able to increase the levels of ROS, leading to increased lipid peroxidation, mitochondrial membrane depolarization and reduction of lysosomal integrity in these fungi [135]. Another relevant target for essential oils is ergosterol, a compound present in the fungal cell membrane whose biosynthesis can be altered by disturbing sterol biosynthetic pathways. Importantly, the absence or reduced presence of ergosterol in fungal membranes results in osmotic and metabolic instability of the fungal cell, compromising reproduction and infectious activity [136]. Indeed, it was shown that *Thymus vulgaris* essential oil and its main compound, thymol, were effective against *Rhizopus oryzae* due to their interaction with ergosterol, thus supporting their use in the management of mucormycosis [124].

## 5. Translation to the Clinic: Limitations and Future Perspectives

Studies comparing different treatments for respiratory mycosis, namely cryptococcal meningitis, candidemia, endemic mycoses, and invasive aspergillosis, are among the most cited clinical trials and are very relevant for the development of treatment guidelines for these infections [137]. Nevertheless, regarding essential oils as anti-infective agents, clinical trials are lacking. Moreover, essential oils present several features such as hydrophobicity, instability, high volatility, and possible toxicity that compromise their use. Therefore, to overcome these limitations, encapsulation resorting to delivery systems, namely lipid-based carriers, have been developed to stabilize these compounds, improve the shelf-life of the formulated products and prolong the biological effect of the active molecules [138]. For example, *Lavandula angustifolia* essential oil encapsulated in liposomes was effective against persistent biofilms of *Candida auris* [78] while *Lippia sidoides* essential oil encapsulated in nanostructured lipid carriers showed anti-*Candida auris* potential and low toxicity, suggesting a new strategy to overcome multidrug-resistant pathogens [79]. On the other hand, due to their volatility, essential oils can easily reach the upper and lower parts of the respiratory tract via active or passive inhalation. In the first case, an inhalation device is needed for the patients to directly inhale the volatile compounds whereas in the latter, heating, vaporization or ventilation is used to deliver these compounds to the environment [139]. Interestingly, over the last years, several patents on portable inhalation devices have been registered and have been shown to be suitable delivery systems for these volatile compounds. Another possible form of administration resorts to patches, the volatiles being released to the skin and/or inhaled by the patient [140].

## 6. Final Remarks

The present review provides, for the first time, an updated compilation of relevant information on the antifungal potential of essential oils and their volatile compounds on respiratory mycosis. Overall, our bibliographic search showed that the majority of the studies are performed on strains involved in opportunistic infections, namely aspergillosis, candidiasis and cryptococcosis, with the present review focusing on strains involved in life-threatening invasive diseases such as *Aspergillus fumigatus* and *Cryptococcus neoformans* and relevant emergent strains such as *Candida auris*. Moreover, it is quite evident that essential oils rich in phenolic compounds, namely thymol and carvacrol, are very effective, and therapeutic improvements can be achieved by combining essential oils and/or their volatile compounds with conventional antifungal drugs. Furthermore, several administration strategies and devices have been designed to effectively deliver these volatile compounds but clinical translation still requires in vivo validations and human trials to confirm the efficacy and tolerability of these extracts/compounds in respiratory mycosis.

## Figures and Tables

**Table 1:** Causal agents of respiratory mycosis.

Fungi	Disease	Source of Infection	Pathological Manifestations	Diagnosis	Antifungals
***Endemic***					
*Blastomyces dermatitidis*	Blastomycosis	Soil	Mild flu-like symptoms; chronic cutaneous disease with subcutaneous lesions on the face and hands	Microscopic observation of sputum samples; urine antigen test; enzyme immunoassay	Amphotericin B, ketoconazole
*Coccidioides immitis*	Coccidioidomycosis (valley fever)	Soil	Granulomatous lesions on the face and nose; meningitis in severe cases	Serological tests	Amphotericin B
*Histoplasma capsulatum*	Histoplasmosis	Soils with bird or bat droppings	Fever, headache, and weakness with some chest discomfort	Chest X-ray; cultures grown on fungal selective media; direct fluorescence antibody and Giemsa staining	Amphotericin B, ketoconazole, itraconazole
*Paracoccidioides* sp.	Paracoccidioidomycosis	Soil near armadillo burrows	Adults: affects lungs and causes lesions in the mouth and throat; Children: swollen lymph nodes and skin lesions	Chest X-ray, biopsy for fungal culture or to be examined under the microscope and blood tests	Itraconazole and amphotericin B; trimethoprim/sulfamethoxazole
*Talaromyces marneffei* (formerly *Penicillium marneffei*)	Talaromycosis (formerly Penicilliosis)	Plants and farmed animals	Fever, weight loss, hepatosplenomegaly, lymphadenopathy, skin lesions	Microscopy, histology, and culture	Amphotericin B or voriconazole followed by itraconazole
*Sporothrix schenckii*	Sporotrichosis (rose gardener’s disease)	Soil, *Sphagnum* moss, rose bushes and hay	Cutaneous nodules that spread and break down into abscesses and ulcers, with rare pulmonary involvement	Culture	Itraconazole, amphotericin B
***Opportunistic***					
*Aspergillus fumigatus*	Aspergillosis	Soils and organic debris	Asthma-like allergic reactions; shortness of breath, wheezing, coughing, runny nose and headaches	Chest X-ray; microscopic examination of tissue and respiratory fluid samples	Itraconazole, voriconazole
*Candida* spp.	Candidiasis	Skin and inside the body	Localized or diffuse pneumonia, nodular lesions, abscesses, and empyema	Isolation of the organism from lung tissue samples	Fluconazole (milder cases), amphotericin B deoxycholate, lipid formulations
*Cryptococcus neoformans*	Cryptococcosis	Soil, pigeon guano and tropical and subtropical trees	Fever, fatigue, and a dry cough; when spreading to the brain causes meningitis (headaches, sensitivity to light, and confusion)	Microscopic examination of lung tissues or cerebrospinal fluids	Amphotericin B combined with flucytosine followed by fluconazole for up to 6 months
Nonpigmented fungi (other than *Aspergillus* and *Penicillium* or *Zygomycetes*) *	Hyalohyphomycosis	Soil, water or on decomposing organic debris	Lesions from local cutaneous, subcutaneous, corneal, or nasal mucosal disease to disseminated disease involving multiple organs	Culture isolation and/or PCR	Surgical removal with or without azole antifungal therapy
*Rhizopus* spp. and *Mucor* spp.	Mucormycosis (formerly zygomycosis)	Throughout the environment	Fever, cough, chest pain, and shortness of breath	Tissue biopsy specimens	Amphotericin B and surgical debridement removal in superficial infections
Dark melanin-pigmented dematiaceous fungi **	Phaeohyphomycosis	Soil	Sinusitis, subcutaneous nodules or abscesses, keratitis, lung masses, osteomyelitis, mycotic arthritis, endocarditis, brain abscess, and disseminated infection	Examination using Masson-Fontana staining; culture to identify causative species	Surgery and/or itraconazole
*Pneumocystis jirovecii*	*Pneumocystis* pneumonia (PCP)	Person to person through the air	Fever, cough, and shortness of breath	Microscopic examination of tissue and fluid samples from the lungs	Trimethoprim-sulfamethoxazole combination

Includes: * *Acremonium*, *Fusarium*, *Geotrichum*, *Paecilomyces*, *Pseudallescheria*, *Sagenomella*, *Phialosimplex*, *Geosmithia*, *Geomyces*, and *Scedosporium*; ** *Bipolaris*, *Cladophialophora*, *Cladosporium*, *Exophiala*, *Fonsecaea*, *Phialophora*, *Ochronosis*, *Rhinocladiella*, and *Wangiella*.

**Table 2:** Antifungal effects of essential oils against *Aspergillus fumigatus*.

Essential Oil (Family)	Plant Part Used	Main Compounds	Antifungal Effect	Ref
*Achillea millefolium* (Asteraceae)	flowering aerial parts	Sample A: α-asarone (33.3%), α-pinene (17.2%), β-bisabolene (16.6%); Sample B: *trans*-thujone (29.0%), *trans*-crhysanthenyl acetate (15.8%), β-pinene (11.1%)	Broth macrodilution method: MIC = 1.25 µL/mL and MLC > 20 µL/mL (A); MIC = 2.5–5 µL/mL and MLC > 20 µL/mL (B)	[33]
*Acorus calamus* (Acoraceae)	*not identified*	*β*-asarone (80.6%)	Tube-dilution method: MFC = 0.104 ± 0.016 mg/mL	[34]
*Allium hookeri* (Amaryllidaceae)	rhizomes	di-2-propenyl trisulfide (31.8%), diallyl disulfide (28.4%)	Broth macrodilution method: MIC = 32 µg/mL and MFC = 64 µg/mL	[35]
*Apium graveolens* (Apiaceae)	flowering aerial parts	Sample A: neophytadiene (34.6%), phytol isomer (11.8%); Sample B: neophytadiene (45.2%), limonene (24.0%)	Broth macrodilution method: MIC = 0.16–0.32 µL/mL and MLC > 125 µL/mL (A); MIC = 0.64 µL/mL and MLC > 20 µL/mL (B)	[36]
*Artemisia absinthium* (Asteraceae)	leaves	borneol (18.7%), methyl hinokiate (11.9%)	Tube-dilution method: MIC = 91 ± 13 µg/mL	[37]
*Artemisia persica* (Asteraceae)	aerial parts	laciniata furanone E (17.1%), artedouglasia oxide C (13.2%), *trans*-pinocarveol (10.2%)	Broth macrodilution method: MIC = 2.5 µL/mL and MFC = 10 µL/mL	[38]
*Beilschmiedia madang* (Lauraceae)	bark	δ-cadinene (20.5%), α-cubebene (15.6%), α-cadinol (10.6%)	Microdilution method: MIC = 62.5 µg/mL	[39]
	leaf	δ-cadinene (17.0%), β-caryophyllene (10.3%), α-cubebene (11.3%)	Microdilution method: MIC = 250 µg/mL	
*Carum copticum* (Apiaceae)	*not identified*	p-cymene (33.7%), thymol (22.8%), γ-terpinene (21.6%)	Broth macrodilution method: MIC = 144 µg/mL	[40]
*Centaurea solstitialis* (Asteraceae)	aerial parts	germacrene D (15.3%), hexadecanoic acid (26.1%), α-linolenic acid (17.9%)	Microdilution assay: MIC = 1.9 µg/mL	[41]
*Cinnamomum camphora* (Lauraceae)	leaf, branch, wood, root, leaf/branch, leaf/branch/wood	leaf: camphor (93.1%); branch: camphor (53.6%); wood: camphor (53.2%) and 1,8-cineole (19.9%); root: safrole (57.6%) and 1.8-cineole (18.1%); leaf/branch: camphor (53.3%); leaf/branch/wood: (59.5%)	Broth microdilution method: MIC = 312.5 µg/mL (leaf, wood and root); MIC = 156.3 µg/mL (branch and leaf/branch); MIC = 78.1 µg/mL (leaf/branch/wood)	[42]
*Cinnamomum glanduliferum* (Lauraceae)	bark	eucalyptol (65.9%)	Broth microdilution method: MIC = 32.5 µg/mL	[43]
*Cuminum cyminum* (Apiaceae)	aerial parts	*not assessed*	Broth microdilution method: MIC = 0.5 mg/mL Broth macrodilution method: MIC = 0.25 mg/mL	[44]
*Daucus carota* subsp. *carota* (Apiaceae)	flowering and ripe umbels	flowering umbels: α-pinene (37.9%), geranyl acetate (15%); ripe umbels— Sample A: α-pinene (13.0%), geranyl acetate (65.0%); Sample B: β-bisabolene (51.0%), (E)-methyl isoeugenol (10.0%)	Broth macrodilution method—Flowering umbels: MIC = 2.5–5 µL/mL and MLC > 20 µL/mL; Ripe umbels: MIC = 0.64–1.25 µL/mL and MLC > 20 µL/mL (A); MIC = 0.64 µL/mL and MLC > 20 (B)	[45]
*Gallesia integrifolia* (Phytolaccaceae)	fruit	2,8-dithianonane (52.6%), dimethyl trisulfide (15.5%), lenthionine (14.7%)	Modified microdilution method: MFC = 0.02—0.18 mg/mL	[46]
*Juniperus communis* subsp. *alpina* (Cupressaceae)	needles	sabinene (26.2%), α-pinene (12.9%), limonene (10.4%)	Macrodilution broth method: MIC = 2.5 µL/mL and MFC = 10 µL/mL	[47]
*Leptospermum petersonii* (Myrtaceae)	*not identified*	*not assessed*	Broth macrodilution method: MIC and MFC = 0.05%	[48]
*Lippia alba* (Verbenaceae)	stems and leaves	Carvone chemotype: carvone (25.3%), limonene (22.4%), geranial (10.4%); Citral chemotype: geranial (30.5%), neral (23.6%)	Microdilution broth method: MIC > 500 µg/mL (both chemotypes)	[49]
*Lavandula luisieri* (Lamiaceae)	flowering aerial parts	Sample A: α-*trans*-necrodyl acetate (17.4%); Sample B: 1,8-cineole (33.9%), fenchone (18.2%)	Broth macrodilution method: MIC = 0.64 µL/mL and MLC = 10–20 µL/mL (A); MIC = 1.25 µL/mL and MLC = 10 µL/mL (B)	[50]
*Lavandula multifida* (Lamiaceae)	flowering aerial parts	carvacrol (42.8%), *cis*-β-ocimene (27.4%);	Broth macrodilution method: MIC = 0.32 µL/mL and MLC = 0.64 µL/mL	[51]
*Lavandula pedunculata* (Lamiaceae)	aerial parts	Sample A: 1,8-cineole (34.3%); Sample B: camphor (34.0%), 1,8-cineole (25.1%); Sample C: fenchone (44.5%)	Broth macrodilution method: MIC = 2.5 µL/mL and MLC = 10 µL/mL (A); MIC and MLC = 5 µL/mL (B); MIC = 5 µL/mL and MLC = 10 µL/mL (C)	[52]
*Lavandula stoechas* (Lamiaceae)	aerial parts	fenchone (37.0%), camphor (27.3%)	Broth macrodilution method: MIC = 1.25 µL/mL and MLC ≥ 20µL/mL	[53]
*Lavandula viridis* (Lamiaceae)	aerial parts	1,8-cineole(34.5%), camphor (13.4%), α-pinene (9.0%), linalool (7.9%)	Broth macrodilution method: MIC = 2.5 µL/mL and MLC = 5–10 µL/mL	[54]
*Melaleuca alternifolia* (Myrtaceae)	*not identified*	*not assessed*	Broth microdilution method: MIC = 0.06–0.12%	[55]
*Myrtus communis* (Myrtaceae)	leaves and flowers	Sample A: α-pinene (50.8%), linalool (14.8%), 1,8-cineole (13.3%); Sample B: α-pinene (33.6%), linalool (14.8%), 1,8-cineole (13.3%)	Broth macrodilution method: MIC = 2.5 mg/mL and MLC > 10 mg/mL (both samples)	[56]
*Nigella sativa* (Ranunculaceae)	aerial parts	*not assessed*	Broth microdilution method: MIC = 2 mg/mL Broth macrodilution method: MIC = 1.5 mg/mL	[44]
*Piper flaviflorum* (Piperaceae)	leaf	(E)-nerolidol (40.5%), β-caryophyllene (14.6%)	Broth microdilution method: MIC = 256 µg/mL and MLC = 1024 µg/mL	[57]
*Ruta angustifolia* (Rutaceae)	flowering aerial parts	2-undecanone (82.5%), 2-decanone (10.0%)	Agar dilution method: MIC < 3.5 µg/mL	[58]
*Ruta chalepensis* (Rutaceae)	flowering aerial parts	2-nonanone (32.8%), 2-undecanone (32.6%), 1-nonene (14.0%)	Agar dilution method: MIC = 6.2–7.4 µg/mL	
*Ruta graveolens* (Rutaceae)	flowering aerial parts	2-undecanone (55.4%), nonanone (21.6%)	Agar dilution method: MIC < 3.5 µg/mL	
*Ruta tuberculata* (Rutaceae)	flowering aerial parts	piperitone (13.6%), *trans*-p-menth-2-en-1-ol (13.1%), *cis*-piperitol (12.3%), *cis*-p-menth-2-en-1-ol (11.2%)	Agar dilution method: MIC < 4.5 µg/mL	
*Satureja thymbra* (Lamiaceae)	aerial parts	thymol (57.3%), γ-terpinene (9.8%), p-cymene (9.8%)	Broth macrodilution method: MIC = 0.32 µL/mL and MLC = 0.64 µL/mL	[59]
*Spondias pinnata* (Anacardiaceae)	fruit peels	furfural (17.1%), α-terpineol (13.1%)	Broth microdilution method: MIC = 16 µg/mL and MFC = 32 µg/mL	[60]
*Thymus villosus* subsp. *lusitanicus* (Lamiaceae)	aerial parts	geranyl acetate (25.0%), terpinen-4-ol (13.5%)	Broth macrodilution method: MIC = 0.64–125 µL/mL and MLC = 2.5–5.0 µL/mL	[61]
*Thymus vulgaris* (Lamiaceae)	*not identified*	thymol (44.7%), γ-terpinene (26.0%), α-cymene (21.2%)	Broth macrodilution method: MIC = 144 µg/mL	[40]
*Ziziphora clinopodioides* (Lamiaceae)	aerial parts	*not assessed*	Broth microdilution method: MIC = 1 mg/mL Broth macrodilution method: MIC = 0.5 mg/mL	[44]

MIC—Minimal Inhibitory Concentration; MBC—Minimal Bacterial Concentration; MFC—Minimal Fungicidal Concentration.

**Table 3:** Antifungal effects of essential oils against *Candida auris*.

Essential Oil (Family)	Plant Part Used	Main Compounds	Antifungal Effect	Mechanism of Action	Ref
*Cinnamomum zeylanicum* (Lauraceae)	bark	*trans*-cinnamaldehyde (66.4%)	Disc diffusion method (diameter of inhibition): 77.37 ± 1.72 mm Broth dilution method: MIC < 0.03% and MFC < 0.03%	Modulation of cell membrane permeability (nuclei acid and protein release); Hemolytic activity	[75]
	leaf	eugenol (62.6%)	Disc diffusion method (diameter of inhibition): 35.40 ± 1.08 mm Broth dilution method: MIC = 0.06% and MFC = 0.25%		
*Eugenia florida* (Myrtaceae)	aerial parts	seline-3,11-dien-6-α-ol (12.9%), eremoligenol (11.0%), γ-elemene (10.7%)	Disc diffusion method (diameter of inhibition): 8 mm		[76]
*Juniperus oxycedrus* subsp. *macrocarpa* (Cupressaceae)	aerial parts	α-pinene (56.6 ± 0.2%), limonene (14.6 ± 0.11%), β-pinene (13.4 ± 0.09%)	Planktonic MIC: 0.02%; Sessile MIC: no biofilm formation		[77]
*Lavandula angustifolia* (Lamiaceae)	*not identified*	linalyl acetate (48.5%), linalool (39.3%)	Growth reduction (0.01%)	Modulation of biofilm related genes	[78]
*Lippia sidoides* (Verbenaceae)	leaves	thymol (68.2%)	Broth microdilution method: Geometric mean MIC = 0.281 mg/mL		[79]
*Myrcia multiflora* (Myrtaceae)	aerial parts	Sample A: α-bulnesene (26.8%), pogostol (21.3%); Sample B: (E)-nerolidol (44.4%); Sample C: (E)-nerolidol (92.2%)	Disc diffusion method (diameter of inhibition): 9 mm (A), 10 mm (B) and 8 mm (C); Broth dilution method: MIC = 3.12 µL/mL (A) and 5 µL/mL (C); MFC > 12.5 µL/mL (A) and >50 µL/mL (C) [*Sample B not assessed*]		[76]
*Thuja plicata* (Verbenaceae)	*not identified*	*not assessed*	Inhibition of the intrinsic rate of growth at 0.09% and 0.39%		[80]
*Thymus mastichina* (Lamiaceae)	flowering cups	linalool (31.9%), α-terpineol (10.0%)	Disc diffusion method (diameter of inhibition): 13.60 ± 1.36 mm	Biofilm inhibition (direct application and vapor phase application)	[81]
*Thymus satureioides* (Lamiaceae)	flowering cups	borneol (29.3%), α-terpineol (15.9%)	Disc diffusion method (diameter of inhibition): 20.00 ± 0.63 mm		
*Thymus vulgaris* (Lamiaceae)	flowering cups	thymol (63.1%), 1,8-cineole (10.0%)	Disc diffusion method (diameter of inhibition): 42.33 ± 3.77 mm		
*Thymus zygis* subsp. *sylvestris* (Lamiaceae)	flowering aerial parts	thymol (26.5%), carvacrol (22.7%)	Disc diffusion method (diameter of inhibition): 28.25 ± 1.0 mm		

MIC—Minimal Inhibitory Concentration; MFC—Minimal Fungicidal Concentration.

**Table 4:** Antifungal effects of essential oils against *Cryptococcus neoformans*.

Essential Oil (Family)	Plant Part Used	Main Compounds	Antifungal Effect	Ref
*Achillea millefolium* (Asteraceae)	flowering aerial parts	Sample A: α-asarone (33.3%), β-bisabolene (16.6%) and α-pinene (17.2%); Sample B: *trans*-thujone (29.0%), *trans*-crhysanthenyl acetate (15.8%) and β-pinene (11.1%)	Broth macrodilution method: MIC = 0.64 µL/mL and MLC = 1.25 µL/mL (A); MIC = 1.25 µL/mL and MLC = 1.25 µL/mL (B)	[33]
*Angelica major* (Apiaceae)	aerial parts	*cis*-β-ocimene (30.4 %), α-pinene (21.8 %)	Broth macrodilution method: MIC = 0.16 µL/mL and MLC = 0.64 µL/mL	[84]
*Apium graveolens* (Apiaceae)	flowering aerial parts	Sample A: neophytadiene (34.6%), γ-himachalene (10.3%); Sample B: neocnidilide (45.2%), limonene (24.0%)	Broth macrodilution method: MIC and MLC = 0.16 µL/mL (A); MIC = 0.32 µL/mL and MLC = 0.64 µL/mL (B)	[36]
*Aristolochia delavayi* (Aristolochiaceae)	aerial parts	(E)-dec-2-enal (52.0%)	Broth microdilution method: MIC = 7.81 µg/mL and MFC = 62.5 µg/mL	[85]
*Artemisia herba-alba* (Asteraceae)	aerial parts	β-thujone (25.1%), α-thujone (22.9%), 1,8-cineole (20.1%), camphor (10.5%)	Broth macrodilution method: MIC = 0.64 mg/mL and MLC = 0.64–125 mg/mL	[86]
*Artemisia judaica* (Asteraceae)	aerial parts	piperitone (30.4%), camphor (16.1%), ethyl cinnamate (11.0%)	Broth macrodilution method: MIC = 0.16 µL/mL and MLC = 0.64 µL/mL	[87]
*Bupleurum rigidum* subsp. *paniculatum* (Apiaceae)	aerial parts	α-pinene (36.0%), β-pinene (26.1%), limonene (10.5%)	Broth macrodilution method: MIC = 72 µg/mL and MLC = 144 µg/mL	[88]
*Chamaecyparis obtusa* (Cupressaceae)	needles and twigs	bicyclo[2.2.1]heptan-2-ol (18.8%), (+)-2-carene (17.4%), sabinene (12.8%)	Broth microdilution method: MIC > 2.18 mg/mL	[89]
*Croton gratissimus* (Euphorbiaceae)	leaves	*not assessed*	Broth microdilution method: MIC = 4 mg/mL	[90]
*Cryptomeria japonica* (Cupressaceae)	needles and twigs	kaur-16-ene (31.5%), sabinene (11.1%),	Broth microdilution method: MIC > 2.18 mg/mL	[89]
*Daucus carota* subsp. *carota* (Apiaceae)	flowering and ripe umbels	Flowering umbels—Sample A: α-pinene (37.9%), geranyl acetate (15.0%); Sample B: carotol (25.1%), β-bisabolene (17.6%); Ripe umbels—Sample A: α-pinene (13.0%), geranyl acetate (65.0%); Sample B: β-bisabolene (51.0%), (E)-methyl isoeugenol (10.0%)	Broth macrodilution method—Flowering umbels: MIC = 0.32–0.64 µL/mL and MLC = 1.25–2.5 µL/mL (A); MIC = 0.32 µL/mL and MLC = 0.32–0.64 µL/mL (B); Ripe umbels: MIC and MLC = 0.64 µL/mL (A); MIC and MLC = 0.64–1.25µL/mL (B)	[45]
	ripe umbels with seeds	geranyl acetate (29.0%), α-pinene (27.2%)	Broth macrodilution method: MIC and MLC = 0.16 µL/mL	[91]
*Distichoselinum tenuifolium* (Apiaceae)	ripe umbels	myrcene (84.6%)	Broth macrodilution method: MIC = 0.32–0.64 µL/mL and MLC = 0.64 µL/mL	[92]
*Foeniculum vulgare* (Apiaceae)	umbels and fruits	E-anetol (47.0%), α-phellandrene (11.0%), α-pinene (10.1%), fenchone (10.8%)	Broth macrodilution method: MIC = 0.32 µL/mL and MLC = 0.64 µL/mL	[93]
*Hirtellina lobelia* (Asteraceae)	aerial parts	α-bisabolol (34.5%), fokienol (12.0%)	Broth microdilution method: MIC = 128 µL/mL	[94]
*Hyptis crenata* (Lamiaceae)	aerial parts	borneol (17.8%), 1,8-cineol (15.6%)	Broth microdilution method: MIC = 62.5 µg/mL and MFC = 125 µg/mL	[95]
*Juniperus communis* subsp. *alpina* (Cupressaceae)	needles	sabinene (26.2%), α-pinene (12.9%)	Broth macrodilution method: MIC = 1.25 µL/mL and MFC = 1.25 µL/mL	[47]
*Lavandula luisieri* (Lamiaceae)	flowering aerial parts	Sample A: α-*trans*-necrodyl acetate (17.4%); Sample B: 1,8-cineole (33.9%), fenchone (18.2%)	Broth macrodilution method: MIC and MLC = 0.64 µL/mL (A); MIC = 0.64 µL/mL and MLC = 0.64–1.2 µL/mL (B)	[50]
*Lavandula multifida* (Lamiaceae)	flowering aerial parts	carvacrol (42.8%), cis-β-ocimene (27.4%)	Broth macrodilution method: MIC = 0.16 µL/mL and MLC = 0.32 µL/mL	[51]
*Lavandula pedunculata* (Lamiaceae)	aerial parts	Sample A: 1,8-cineole (34.3%); Sample B: camphor (34%), 1,8-cineole (25.1%); Sample C: fenchone (44.5%)	Broth macrodilution method: MIC and MLC = 1.25 µL/mL (A); MIC and MLC = 0.32–0.64 µL/mL (B); MIC = 1.25 µL/mL and MLC = 1.25–2.5µL/mL (C)	[52]
*Lavandula viridis* (Lamiaceae)	aerial parts	1,8-cineole(34.5%), camphor (13.4%), α-pinene (9.0%), linalool (7.9%)	Broth macrodilution method: MIC = 0.64 µL/mL and MLC = 0.64 µL/mL	[54]
*Melaleuca alternifolia* (Myrtaceae)	*not identified*	terpinen-4-ol (42.4%), γ-terpinene (20.7%)	Broth microdilution method: MIC = 0.06–0.2%	[96]
*Mentha x piperita* (Lamiaceae)	leaves	menthol (41.7%), menthone (21.8%)	Broth microdilution method: MIC = 0.06–0.125% and MFC = 0.06–0.125%	[97]
*Mentha pulegium* (Lamiaceae)	aerial parts	pulegone (86.2%)	Broth macrodilution method: MIC = 0.64 mg/mL and MLC = 1.25 µL/mL	[98]
*Mentha spicata* (Lamiaceae)	aerial parts	carvone (62.9%)	Broth macrodilution method: MIC = 0.32 µL/mL and MLC = 0.64–1.25 µL/mL	
*Mesembryanthemum edule*(Aizoaceae)	leaves	tetradecamethylcycloheptasiloxane (13.6%), phytol (12.4%)	Broth microdilution method: MIC = 0.08 mg/mL	[99]
*Mitracarpus frigidus* (Rubiaceae)	aerial parts	linalool (29.9%), eugenol acetate (15.9%)	Broth microdilution method: MIC = 8 µg/mL	[100]
*Myrtus communis* (Myrtaceae)	dried leaves and flowers	Sample A: α-pinene (50.8%), 1,8-cineole (21.9%); Sample B: α-pinene (33.6%), linalool (14.8%), 1,8-cineole (13.3%)	Broth macrodilution method: MIC = 0.64 mg/mL and MLC = 0.64–1.25 mg/mL (A); MIC and MLC = 0.64 mg/mL (B)	[56]
*Myrtus nivellei* (Myrtaceae)	aerial parts (10 samples)	1,8-cineole (37.5%), limonene (25.0%)	Broth macrodilution method: MIC = 0.16 µL/mL and MLC = 0.32 µL/mL	[101]
*Oenanthe crocata* (Apiaceae)	aerial parts	*trans*-*β*-ocimene (31.3%), sabinene (29.0%), *cis*-*β*-ocimene (12.3%)	Broth macrodilution method: MIC = 0.16 µL/mL and MLC = 0.32 µL/mL	[102]
*Pinus densiflora* (Pinaceae)	needles and twigs	β-phellandrene (16.7%), (-)-α-pinene (14.9%), 1-β-pinene (10.5%), α-fenchyl acetate (10.3%)	Broth microdilution method: MIC = 0.545 mg/mL	[89]
*Pistacia x saportae* (Anacardiaceae)	aerial parts	α-pinene (30.3%), (Z)-β-ocimene (26.7%), (E)-β-ocimene (11.1%)	Broth macrodilution method: MIC = 0.32 mg/mL and MLC = 0.64–1.25 mg/mL	[103]
*Pistacia lentiscus* (Anacardiaceae)	aerial parts	terpinen-4-ol (25.2%), α-phellandrene (11.9%), β-phellandrene (10.2%), γ-terpinene (10.1%)	Broth macrodilution method: MIC = 0.32 mg/mL and MLC = 0.64 mg/mL	
*Pistacia terebinthus* (Anacardiaceae)	aerial parts	terpinolene (35.2%), α-pinene (35%)	Broth macrodilution method: MIC = 1.25 mg/mL and MLC = 2.5 mg/mL	
	leaves	Sample A: α-pinene (62.4%), β-pinene (12.1%); Sample B: terpinolene (35.2%), α-pinene (35.0%)	Broth macrodilution method: MIC = 0.32 µL/mL and MLC = 0.64 µL/mL (A); MIC = 1.25 µL/mL and MLC = 2.5 µL/mL (B)	[104]
*Protium amazonicum* (Myrtaceae)	oleoresin	δ-3-carene (47.9%)	Broth microdilution method: MIC = 0 156 μg/mL	[105]
*Santiria trimera* (Burseraceae)	bark	α-pinene (66.6%), β-pinene (20.0%)	Agar dilution method: MIC < 0.71 μL/mL	[106]
*Santolina impressa* (Asteraceae)	flowering aerial parts	β-pinene (22.5%), 1,8-cineole (10.0%)	Broth macrodilution method: MIC = 0.27 mg/mL	[107]
*Santolina insularis* (Asteraceae)	aerial parts	β-phellandrene (22.6%), myrcene (11.4%)	Broth macrodilution method: MIC = 0.13 mg/mL	[108]
*Satureja thymbra* (Lamiaceae)	aerial parts	thymol (57.3%), γ-terpinene (9.8%), β-caryophyllene, p-cymene (9.8%)	Broth macrodilution method: MIC = 0.16 µL/mL and MLC = 0.32 µL/mL	[59]
*Smyrnium olusatrum* (Apiaceae)	fruiting umbels	Sample A: β-phellandrene (42.7%); Sample B: acetoxyfurano-4(15)-eudesmene (17.6%)	Broth macrodilution method: MIC = 0.32 µL/mL and MLC = 0.64 µL/mL (A); MIC and MLC = 0.64 µL/mL (B)	[109]
*Tanacetum vulgare* (Asteraceae)	flowering aerial parts	1,8-cineole (18.2%), myrtenol (10.3%)	Broth macrodilution method: MIC = 0.16 µL/mL and MLC = 0.16–0.32 µL/mL	[110]
*Teucrium scordium* subsp. *scordioides* (Lamiaceae)	aerial parts	germacrene D (25.1%), δ-cadinene (12.9%), alloaromadendrene (11.3%)	Broth macrodilution method: MIC = 0.32 µL/mL and MLC = 0.32 µL/mL	[111]
*Thapsia villosa* (Apiaceae)	aerial parts	limonene (57.5%), methyleugenol (35.9%)	Broth macrodilution method: MIC and MFC = 0.16 µL/mL	[112]
*Thymus camphoratus* (Lamiaceae)	flowering aerial parts	1,8-cineole (15.5%), α-pinene (12.7%)	Broth macrodilution method: MIC = 0.14 mg/mL and MLC = 0.28 mg/mL (both oils)	[113]
*Thymus carnosus* (Lamiaceae)	flowering aerial parts	borneol (29.0%), camphene (19.5%)		
*Thymus villosus* subsp. *lusitanicus* (Lamiaceae)	aerial parts	terpinen-4-ol (13.5%), geranyl acetate (25%)	Broth macrodilution method: MIC = 0.16 µL/mL and MLC = 0.16–0.32 µL/mL	[61]
*Vitex rivularis* (Lamiaceae)	leaves and flowers	Sample A: germacrene D (12.6%); Sample B: germacrene D (20.6%)	Broth macrodilution method: MIC = 0.64–1.25 µL/mL and MLC = 2.5–5 µL/mL (A); MIC = 1.25 µL/mL and MLC = 5–10 µL/mL (B)	[114]
*Ziziphora tenuior* (Lamiaceae)	aerial parts	pulegone (46.8%), p-menth-3-en-8-ol (12.5%)	Broth macrodilution method: MIC = 0.16 µL/mL and MLC = 0.64 µL/mL	[115]

MIC—Minimal Inhibitory Concentration; MFC—Minimal Fungicidal Concentration; MLC—Minimal Lethal Concentration.

**Table 5:** In vitro antifungal effects of volatile compounds against respiratory fungi.

Fungal Strain Tested	Essential Oil (Family)	Plant Part Used	MainCompounds	Antifungal Effect	Ref
*Rhizopus oryzae*	*Thymus vulgaris* (Lamiaceae)	*not identified*	*not assessed*	Disc diffusion method (diameter of inhibition): 32 mm; Broth microdilution method: MIC = 256–512 μg/mL; MFC = 512–1024 μg/mL (several strains tested)	[124]
*Paracoccidioides brasiliensis*	*Schinus molle* (Anacardiaceae)	leaves	β-pinene (25.2%), epi-α-cadinol (21.3%), α-pinene (18.7%), myrcene (11.5%)	Broth microdilution method: MIC and MLC = 39.06 μg/mL	[125]
*Sporothrix schenckii*	*Origanum majorana* (Lamiaceae)	*not identified*	1.8-cineole (20.9%), terpeninen-4-ol (20.4%)	Broth microdilution method: MIC and MLC ≤ 2.25 mg/mL	[126]
